# To look or not to look during vaccination: A pilot randomized trial

**DOI:** 10.1080/24740527.2017.1412254

**Published:** 2018-01-05

**Authors:** Priyanjali Mithal, Pamela Simmons, Tessa Cornelissen, Horace Wong, Rebecca Pillai Riddell, C. Meghan McMurtry, Lisa Burry, Derek Stephens, Anna Taddio

**Affiliations:** a Leslie Dan Faculty of Pharmacy, University of Toronto, Toronto, Ontario, Canada; b Health and Wellness Centre, University of Toronto, Toronto, Ontario, Canada; c Department of Psychology, York University, Toronto, Ontario, Canada; d Department of Psychology, University of Guelph, Guelph, Ontario, Canada; e Child Health Evaluative Services, The Hospital for Sick Children, Toronto, Ontario, Canada

**Keywords:** vaccination, pain, fear, needle, looking preference

## Abstract

**Background:**

Clinicians commonly advise patients to look away from the needle during vaccinations; however, this recommendation is not evidence based.

**Aim:**

The aim of this study was to determine whether looking at the needle versus looking away affects pain and fear during vaccinations in adults.

**Methods:**

This was a pilot randomized two-group parallel trial with university students receiving influenza vaccinations. Participants were stratified according to their initial needle-looking preference and randomly assigned to either look at versus away from the needle. Participants self-reported their pain and fear during vaccination.

**Results:**

Of the 184 subjects who agreed to participate, 160 were enrolled; 66% were female. A three-way analysis of variance (ANOVA; Looking allocation assignment × Looking preference × Sex) revealed a significant main effect of looking allocation assignment on fear (*P* = 0.025); those who were randomized to look had higher fear scores than those who were randomized to look away. There was also a significant main effect of looking preference on fear (*P* < 0.001); those who preferred to look away had higher fear scores than those who preferred to look. There was no evidence of an effect of looking allocation assignment or looking preference on pain. There was a significant main effect of sex on fear and pain, with females reporting higher pain and fear scores than males (*P* = 0.017 and *P* = 0.001, respectively). There were no significant interactions.

**Conclusion:**

These preliminary findings suggest that advising individuals to look away from the needle reduces fear. A larger trial including more individuals and a different population is recommended to confirm the results.

## RÉSUMÉ

**Contexte**: Les cliniciens conseillent habituellement aux patients de ne pas regarder l’aiguille pendant la vaccination; toutefois, cette recommandation ne s’appuie pas sur des données probantes.

**But**: Déterminer si le fait de regarder l’aiguille ou de ne pas la regarder influence la douleur et la peur pendant la vaccination chez les adultes.

**Méthodes**: Un essai pilote randomisé avec deux groupes parallèles a été mené auprès d’étudiants universitaires devant recevoir un vaccin contre l’influenza. Les participants ont été stratifiés selon leur préférence initiale pour regarder l’aiguille ou ne pas la regarder. Ils ont ensuite été répartis de façon aléatoire entre un groupe assigné à regarder l’aiguille et un groupe assigné à ne pas la regarder. Les participants ont autodéclaré leur douleur et leur peur pendant la vaccination.

**Résultats**: Parmi les 184 sujets ayant accepté de participer à l’étude, 160 y ont été inscrits, dont 66 % de femmes. Une ANOVA à trois critères (ne pas regarder l’aiguille x préférence pour regarder ou ne pas regarder l’aiguille x sexe) a révélé un effet significatif de l’assignation à  regarder l’aiguille sur la peur (p = 0,025); ceux qui ont été randomisés pour regarder l’aiguille ont obtenu un score plus élevé en ce qui concerne la peur que ceux qui ont été randomisés pour ne pas la regarder. Un effet significatif de la préférence pour regarder sur la peur également été observé (p < 0,001) : ceux qui préféraient ne pas regarder l’aiguille ont obtenu un score plus élevé en ce qui concerne la peur que ceux qui préféraient regarder. Aucune preuve d’un effet de l’assignation à regarder ou de la préférence pour regarder sur la douleur n’a été relevée. Un effet significatif du sexe sur la peur et sur la douleur a été observé, les femmes ayant déclaré des niveaux de douleur et de peur plus élevés que les hommes (p = 0,017 et p = 0,001, respectivement). Aucune interaction significative n’a été observée.

**Conclusion**: Ces résultats préliminaires suggèrent que le fait de conseiller aux personnes de ne pas regarder l’aiguille réduit la peur. Un essai plus vaste mené auprès d’un plus grand nombre d’individus et d’une population différente est recommandé afin de confirmer les résultats.

**Trial Registration No**. NCT02937428.

## Introduction

Vaccinations prevent morbidity and mortality from infectious diseases.^1^ However, approximately 25% of adults are afraid of needles, with anticipated needle pain being a significant source of this fear.^2^ This potential for pain and fear presents an important barrier to vaccination uptake, resulting in one out of 12 adults either delaying or refusing vaccinations entirely.^3,4^ Mitigating pain and fear may lead to increased vaccination compliance and favorably impact long-term health outcomes, for both individuals and communities.^5–9^ Many evidence-based strategies are available to help reduce needle pain and fear and combat noncompliance, but they are underutilized in clinical practice (e.g., topical anesthetics).^8^ In addition, the majority of these interventions are targeted toward children.^9–12^ There is a need to find feasible interventions for adults undergoing vaccination.

One simple intervention used by some clinicians to reduce fear and pain involves advising individuals to look away from the needle during injection.^10^ To our knowledge, there are no randomized trials that have determined the effectiveness of this intervention with respect to reducing fear or pain during vaccination. In four nonclinical and one clinical observational study performed to date, conflicting results were obtained for self-reported pain after asking healthy adults to either look at versus look away during a painful stimulus.^10,13–16^ Higher pain scores during looking have been explained as possibly due to increasing autonomic nervous system activity in the individual.^13,14^ Conversely, lower pain scores during looking have been explained as possibly preventing an individual’s imagination from conjuring up a more traumatic experience than reality.^10^ This evidence base did not include vaccinations. The nonclinical studies incorporated pain ratings from electrical, laser, and thermal stimuli, whereas the clinical study examined venipuncture.^10,13–16^ The effects of looking at or looking away from different medical procedures involving needles may have different fear- and pain-inducing components.^9^ It is important to explore the effects of this intervention during vaccinations specifically.

The pain and fear experience may be influenced by personal needle-looking preferences. In the only observational study carried out in a clinical setting, 73% of 192 adult participants spontaneously looked away during venipuncture. These participants reported higher pain scores than those who preferred to look at the needle.^10^ According to the author, it is possible that those who naturally look away have higher pain scores because the propensity to look away may be a marker of low pain tolerance.^10^ In those who prefer to look, repeated pairings of looking at the needle during needle procedures may have a desensitizing effect. A mismatch between personal preference and clinician recommendation may negatively impact the person’s pain and fear experience.

It is possible that an individual’s preference to look at or look away from noxious stimuli is based on underlying coping style and may impact an individual’s pain and fear experience. The literature on coping is complex, with numerous ways to conceptualize different strategies.^17–19^ One conceptualization distinguishes between *sensitizers* versus *repressors*, who are characterized by approach versus avoidance behaviors, respectively.^18,19^ Sensitizers tend to actively seek out information concerning the nature of the stressful situation, whereas repressors avoid information or use coping strategies such as denial.^18,19^ In the context of needle procedures, sensitizers would prefer to watch the needle and learn about each step of the procedure, whereas repressors would prefer to look away from the procedure and/or focus on something else. The success of these coping strategies likely depends on what is being attended to/what sensitizers are focusing on (e.g., sensations vs. emotions).^19^ To date, however, no research has examined the complex interplay between clinical recommendations for looking and one’s own looking preference as coping strategies and how this interaction may have an impact on pain and fear perception.

We undertook a pilot randomized trial to explore the impact of being instructed to look at the needle to reduce pain and fear versus looking away, while accounting for looking preferences of individuals. The specific objectives were to examine feasibility, acceptability, and fidelity and estimate effects to inform a larger randomized control trial.

## Materials and methods

### Population and setting

We conducted a pilot randomized, two-group, parallel, open trial. The setting was the University of Toronto Health and Wellness Centre. Eligible participants included any student (18+ years old) enrolled in the undergraduate and graduate programs at the Leslie Dan Faculty of Pharmacy who consented to (1) receiving influenza vaccinations and (2) being randomized to either look at the needle or look away from it during vaccination. Study data collection was conducted between October 24, 2016, and January 5, 2017.

### Materials

A questionnaire administered by a research assistant (RA) was used to collect demographic information (e.g., sex, age, ethnicity) and looking preference. Looking preference was ascertained using the question, “When getting injections, do you usually prefer to look at or look away from the needle?”

Postvaccination, an RA administered two 11-point Verbal Numerical Rating Scales (VNRS)^8,20^ to collect pain and fear scores. The following two questions were asked: (1) “On a scale of 0 to 10, where 0 is no pain, and 10 is the most pain possible, how would you rate your pain during the needle?” and (2) “On a scale of 0 to 10, where 0 is no fear, and 10 is the most fear possible, how fearful were you during the needle?” In addition, postvaccination, participants reported their needle-looking preferences for future vaccinations. This was based on responses of the participants to the question, “What would you prefer to do for your future vaccinations? [look, look away, no preference].” The postvaccination questionnaire also included a compliance checkbox whereby an RA checked off either “yes” or “no” to the question: “Was the participant compliant with the study instructions?”

### Consenting process

Posters, e-mails, and class announcements were used to introduce the study to all registered students (total population *N* = 1109). On-site recruitment was conducted for 3 weeks (from October 3, 2016, to October 21, 2016) on weekdays from 9 am to 5 pm, whereby interested individuals approached the study coordinator to enroll. Written informed consent was obtained from all participants. As per routine practice, appointments were scheduled for vaccinations. A confirmation e-mail reminder was sent. The study was approved by the University Research Ethics Board. The study was registered on www.clinicaltrials.gov (NCT02937428).

### Randomization and allocation concealment

An RA not directly associated with trial execution created a randomization code using a computerized random number generator. A separate code was used according to individual looking preference; that is, patients were stratified based on baseline preference to either look at or look away from the needle. Within each stratum, individuals were randomized to either look at or look away from the needle during the vaccine injection using a 1:1 allocation ratio. Thus, there were four study groups: (1) prefer to look away and randomized to look; (2) prefer to look away and randomized to look away; (3) prefer to look and randomized to look; and (4) prefer to look and randomized to look away. Sequentially numbered opaque sealed envelopes were prepared for each stratum. This RA did not have any other involvement in the trial. On the day of the vaccination, a different RA opened the next envelope to reveal the allocation group of the participant immediately before vaccination.

### Study procedures

Two registered nurses at the Health and Wellness Centre administered all vaccine injections. Both nurses underwent training prior to study execution to ensure understanding of the protocol and consistency in injection techniques used for vaccinations. Training included scripts for interactions with participants (described further below). Three RAs were involved in data collection; one was responsible for greeting and registering participants, the second collected baseline information (demographics and needle-looking preference) and opened the concealed envelope containing the allocation group, and the third collected postvaccination data, which included pain and fear ratings, compliance with the intervention, and future needle-looking preference.

All vaccinations occurred in private rooms with separate entrances and exits. The nurse provided standard information about the influenza vaccine and asked the participant questions in accordance to usual practice at the clinic. All participants sat upright on a chair, with their elbow resting on the armrest. The participant’s nondominant arm was swabbed with alcohol (70% isopropyl alcohol, Healthcare, Medical Mart, Mississauga, Ontario, Canada). The nurse asked the participant to “relax the arm and let it go all loose and jiggly.” Then the participant was asked to “look directly at the needle” or “look away from the needle in the other direction,” according to group allocation. The nurse said, “Here I go …” right before injecting the vaccine. All participants received 0.5 mL of Fluviral (Sanofi, GlaxoSmithKline, Mississauga, Ontario, Canada) intramuscularly, without prior aspiration, using a 0.5 mm × 25 mm needle (BD Eclipse, Becton, Dickinson and Company, Rantoul, Illinois, USA). After injection, a band aid was applied and the nurse said, “We’re all done.” The RA asked participants about their level of pain and fear during the vaccination. Compliance with the intervention was noted and participants were asked about their future needle-looking preferences.

### Study outcomes

#### Primary outcomes

- Pain: Self-reported pain was assessed using an 11-point VNRS,^17^ where 0 = *no pain* and 10 = *most pain possible* immediately after the vaccination (<5 min).
- Fear: Self-reported fear was assessed using an 11-point VNRS,^8^ where 0 = *no fear* and 10 = *most fear possible* immediately after the vaccination (<5 min).

#### Secondary outcomes

- Feasibility: The first feasibility criterion was achieving a minimum overall recruitment rate of 15% (*n* = 166) over a 6- to 8-week period (study timeline). This was based on an estimate of 30% of the eligible population (*N* = 1109) getting vaccinated and a 50% enrollment rate. The second criteria for feasibility was an assumption of an equal split (50:50) in the number of people who preferred to look at or away from the needle at baseline (to allow for an equal number of participants in each stratum).
- Acceptability: Acceptability of the intervention was based on three criteria: First, a rate of >75% of participants completing the trial; second, a duration of appointment of <15 min; and third, the looking preference of participants for future vaccinations.
- Fidelity: Fidelity of the intervention was based on a rate of >75% of participants being compliant with the instruction to look at the needle or look away during the vaccination.

### Sample size calculation and statistical analysis

In the absence of any prior data to guide effect size estimates, the sample size was arbitrarily set to *n* = 40 per group (total, *n* = 80), according to Hertzog.^21^ Because two strata were included, the sample size was doubled (i.e., *n* = 80 × two strata, or total, *n* = 160), assuming a 50:50 split.

Demographic characteristics (i.e. age, sex, ethnicity) were compared between groups using a *t*-test and χ2 test, as appropriate. Two three-way analyses of variance (ANOVAs; Looking allocation assignment × Looking preference × Sex) were used to examine pain and fear scores. Looking allocation assignment included two levels (randomized to look at versus randomized to look away). Similarly, looking preference included two levels (participant preference to look at versus preference to look away), and sex included two levels (male versus female). Interactions were removed from the model if nonsignificant. Sex was included as an independent factor in the model because previous studies demonstrate sex differences in both pain and fear, with females reporting more pain and fear than males.^19–22^ Q-Q plots were examined to rule out departure from normality in the residuals of the model. There were no observable departures from normality. The Pearson correlation coefficient was used to examine the relationship between pain and fear scores during vaccination.

## Results

### Participant flow

**Figure 1.**
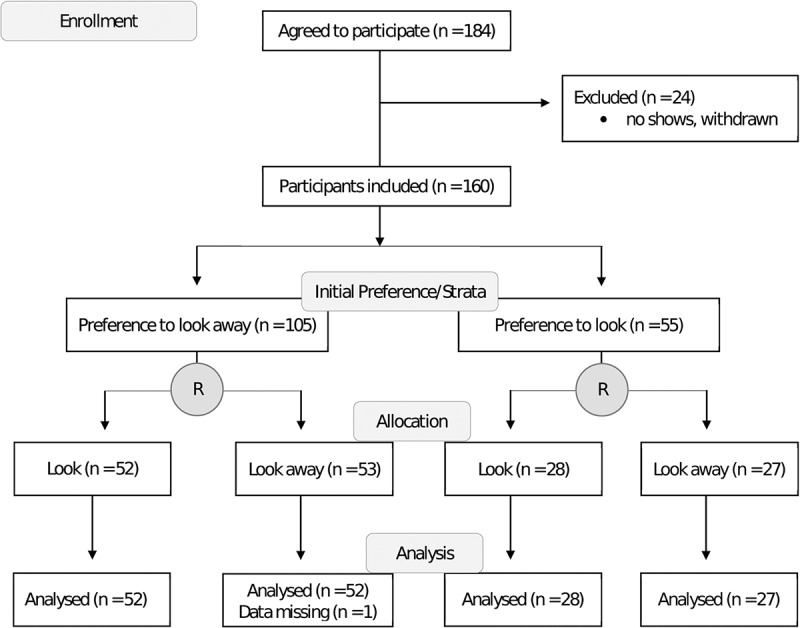
CONSORT participant flow diagram.

Data collection was conducted between October 24, 2016, and January 5, 2017. Altogether, 184 were enrolled, of whom 24 (13%) did not attend the appointment, leaving 160 participants. Fifty-five (34%) self-identified at baseline as preferring to look at the needle; the remaining 105 (66%) preferred to look away from the needle. Figure 1 shows the participant flow during the trial.

### Participant characteristics

There were no statistically significant differences (*P* > 0.05) in demographic characteristics among the four groups (Table 1).

**Table 1.** Participant characteristics (*n* *=* 160).^a^

	Preference to look, randomized to look (*n* = 28)	Preference to look, randomized to look away (*n* = 27)	Preference to look away, randomized to look (*n* = 52)	Preference to look away, randomized to look away (*n* = 53)	*P* value^b^
Age, years	23.8 (3.8)	23.7 (2.2)	23.1 (1.8)	23.2 (2.1)	0.487
Sex, female	14 (50)	17 (63)	38 (73)	37 (70)^c^	0.194
Ethnicity, Asian	23 (82)	16 (59)	37 (71)	31 (58)	0.086

^a^Values are mean (SD) or frequency (%).

^b^Chi square test or ANOVA.

^c^Missing data (*n* = 1).

### Primary outcomes

Table 2 presents the means and standard deviations of the pain ratings of males and females, immediately following the vaccination. There were no significant interactions. The analysis demonstrated no evidence of an effect of looking allocation assignment on pain: randomized to look (*n* = 80, mean = 3.09, SD = 1.93), versus randomized to look away (*n* = 79, mean = 2.95, SD = 2.02), F(1,55) = 0.33, *P* = 0.567. There was no evidence of an effect of looking preference on pain: preference to look (*n* = 55, mean = 2.58, SD = 1.67) versus preference to look away (*n* = 104, mean = 3.25, SD = 2.08), F(1,155) = 2.57, *P* = 0.111. There was, however, a significant effect of sex on pain: females (*n* = 105, mean = 3.42, SD = 1.94) versus males (*n* = 54, mean = 2.24, SD = 1.79), F(1,155) = 12.08, *P* = 0.0007.

**Table 2.** Verbal Numerical Rating Scale^a^ pain scores of males and females.^b^

	Randomized to look	Randomized to look away	Row mean (SD)
**Males**			
Preference to look	(*n* = 14)	(*n* = 10)	(*n* = 24)
	2.25 (1.72)	2.0 (1.49)	2.15 (1.60)
Preference to look away	(*n* = 14)	(*n* = 16)	(*n* = 30)
	2.54 (2.10)	2.13 (1.86)	2.32 (1.95)
Column mean (SD)	(*n* = 28)	(*n* = 26)	
	2.39 (1.89)	2.08 (1.70)	
**Females**			
Preference to look	(*n* = 14)	(*n* = 17)	(*n* = 31)
	2.71 (1.59)	3.09 (1.75)	2.92 (1.66)
Preference to look away	(*n* = 38)	(*n* = 36)	(*n* = 74)
	3.75 (1.90)	3.51 (2.17)	3.64 (1.97)
Column mean (SD)	(*n* = 52)	(*n* = 53)	
	3.47 (1.86)	3.38 (2.04)	

^a^Scores range from 0 to 10 (0 = *no pain*, 10 = *most pain possible*) immediately postvaccination.

^b^Values are mean (SD). General Linear Model (GLM) procedure three-way ANOVA results for pain: There was a significant main effect of sex, F(1,155) = 12.08, *P* = 0.0007, not strata, F(1,155) = 2.57, *P* = 0.111, or randomization (looking allocation assignment), F(1,155) = 0.33, *P* = 0.567; see text for details.

Table 3 presents fear ratings of males and females. There were no significant interactions. There was a significant main effect of looking allocation assignment on fear: randomized to look (*n* = 80, mean = 2.81, SD = 2.3) versus randomized to look away (*n* = 79, mean = 2.06, SD = 2.3), F(1,155) = 5.14, *P* = 0.025. There was a significant main effect of looking preference: preference to look (*n* = 55, mean = 1.55, SD = 2.3) versus preference to look away (*n* = 104, mean = 2.91, SD = 2.3), F(1,155) = 11.79, *P* = 0.0008. There was a significant main effect of sex on fear: females (*n* = 105, mean = 2.79, SD = 2.3) versus males (*n* = 54, mean = 1.75, SD = 2.3), F(1,155) = 5.78, *P* = 0.017.

**Table 3.** Verbal Numerical Rating Scale^a^ fear scores of males and females.^b^

	Randomized to look	Randomized to look away	Row mean (SD)
**Males**			
Preference to look	(*n* = 14)	(*n* = 10)	(*n* = 24)
	1.25 (1.01)	1.65 (1.25)	1.42 (1.11)
Preference to look away	(*n* = 14)	(*n* = 16)	(*n* = 30)
	2.36 (1.69)	1.72 (1.71)	2.02 (1.70)
Column mean (SD)	(*n* = 28)	(*n* = 26)	
	1.8 (1.48)	1.69 (1.52)	
**Females**			
Preference to look	(*n* = 14)	(*n* = 17)	(*n* = 31)
	1.64 (2.5)	1.65 (1.66)	1.65 (2.04)
Preference to look away	(*n* = 38)	(*n* = 36)	(*n* = 74)
	3.97 (2.65)	2.53 (2.34)	3.27 (2.59)
Column mean (SD)	(*n* = 52)	(*n* = 53)	
	3.35 (2.79)	2.25 (2.17)	

^a^Scores range from 0 to 10 (0 = *no fear*, 10 = *most fear possible*) immediately postvaccination.

^b^Values are mean (SD). GLM procedure three-way ANOVA results for fear: There was a significant main effect for sex, F(1,155) = 5.78, *P* = 0.017, strata (looking preference), F(1,155) = 11.79, *P* = 0.0008, and randomization (looking allocation assignment), F(1,155) = 5.14, *P* = 0.025; see text for details.

The Pearson correlation coefficient between pain and fear scores was 0.60 (*P* < 0.0001).

### Secondary outcomes

- Feasibility: Altogether, 184 participants were enrolled; the recruitment rate was 17%. The ratio of people who had a baseline preference to look at the needle versus look away from the needle was 66:34 in the sample.
- Acceptability: All (100%) of participants who were randomized stayed in the study (i.e., none of the participants withdrew). The mean (SD) time taken for the entire procedure from baseline questions, to vaccination procedure, to postvaccination questions was 11 min 28 s (±2 min 40 s). Altogether, 74.4% of participants maintained their original looking/not looking preference for future vaccinations.
- Fidelity: One participant (0.6%) was noncompliant with the intervention. The participant had a baseline preference to look away and was randomized to look at the needle. This individual looked away during injection.

## Discussion

To our knowledge, this is the first randomized trial to examine the impact of looking at the needle versus looking away during vaccination on pain and fear in adults. Feasibility, acceptability, and fidelity of the intervention were demonstrated. Looking at the needle versus looking away did not have a significant impact on pain but did significantly impact fear, with those who were told to look at the needle reporting significantly more fear. Similarly, those who initially preferred to look away reported significantly more fear. Females in our study reported significantly more pain and fear than males. There was no evidence of any interactions between the factors evaluated. Thus, there was no evidence that a mismatch between initial preference to look or not look and randomization group impacts pain or fear.

Assessing an individual’s fear during vaccination is important because fear can increase pain perception.^9^ Concerns about fear and pain can also negatively impact future vaccination compliance.^9^ Of note, reported pain did not vary with looking behavior in the present study, but reported fear did. It is possible that the fear experienced by participants was not limited to the fear of pain but included apprehensions about other aspects of the procedure.^9^ It is also possible that no effect was observed for pain due to the small sample size, relatively low levels of fear and pain in our study population, and the specific procedure and vaccine used.^9,10^ Future studies are recommended that examine different populations, procedures, and vaccines.

Studies have shown that females report higher pain and fear than males.^22–25^ Differences in physiological responses to pain and fear have been observed in males and females, including pupil dilation, brain scans, and other neurological parameters.^22,23^ Explanations may include psychosocial factors, hormonal factors, genetic factors, or a combination of all of these.^23^ For instance, it is possible that males are conditioned to report less pain and fear due to differences in reinforcement of expressions of pain and fear in childhood.^23–25^ The results of this study are consistent with this literature—females reported more pain and fear during vaccination than their male counterparts.

The intervention evaluated in the present study demonstrated high feasibility, acceptability, and fidelity. The recruitment rate met our a priori criterion for demonstrating that the study was feasible because we met our recruitment rate of 15% within a 3-week period (faster than the anticipated 6–8 weeks). To account for the 24 participants who did not attend their appointments, additional participants were enrolled for an additional recruitment week. This suggests that enrolling participants at the proposed rate for a similar study in the future is feasible. The ratio of people who had baseline preference to look at the needle versus look away was anticipated to be 50:50; however, the actual split was 34:66, which precluded us from reaching the desired sample size for people with a baseline preference to look at the needle. An uneven split was also reported in a previous observational study by Vijayan et al.^10^ that assessed spontaneous behavior, in which the ratio was 27:73. Future studies will need to account for this difference in recruitment rates if both needle-looking preferences are to be included.

The trial was deemed acceptable based on the low attrition rate (i.e., no dropouts/withdrawals once randomized) and short duration required to implement the intervention (approximately five to six more minutes than usual practice, including all study procedures) per participant. The average time for vaccination including the study procedures was just over 10 min, approximately double the time usually taken in the clinic. This additional time can be built into a future trial. Though compliant with the intervention, the majority of participants reported preferring to undergo future vaccinations using their baseline preference. This should be explored further, including asking participants about their satisfaction with the procedure.

Fidelity of the intervention was demonstrated by compliance of all but one participant with the instructions given by the nurse (i.e., >99% compliance). It was anticipated that those who had initial preference to look away and were randomized to look would find it hard to be compliant with the instructions to look. However, this was not the case in our study, at least as measured by observable behavior.

Limitations of the study include the small sample size and limited diversity in demographic of included participants (i.e., pharmacy students). Health care students, in general, may have a higher tolerance for pain and fear when it comes to vaccination due to a desensitization effect from undergoing more routine vaccinations than the average person.^26^ In addition, nurses, research personnel, and the participants were not blinded to the study objective, which could have introduced bias. Using an open design was considered acceptable because there was no evidence suggesting that one way (i.e., looking at the needle or not looking at it) is better than the other (i.e., bias was minimized by the lack of perceived benefit of one condition over the other).

There are numerous strengths of the study. First, personal needle-looking preference was taken into account prior to randomization. Second, the study used rigorous methodology, including randomization and allocation concealment. Third, the study procedures were standardized in order to reduce performance and detection bias that may have been introduced due to the open nature of this trial. Finally, there were no dropouts, minimizing attrition bias.

## Conclusion

This pilot study suggests that regardless of initial looking preference or sex, telling adults to look away from the needle during vaccination can reduce fear. Alternatively, clinicians can go by the individual’s preference, especially accommodating those who have a strong preference to look away. The results have implications for clinical practice. Simply telling individuals to look away from the needle is an easy, free strategy that requires no training and can help to reduce fear during vaccination. However, given the preliminary nature of the results, we recommend caution interpreting these results and repeating the study using a larger sample size and a different population to further examine the relationship between looking preference and telling individuals to look at versus away from the needle. This is particularly important for individuals who declare a preference to look away and report higher levels of fear. We also recommend further research for the effect of this intervention during other needle procedures.
